# What is the future of treatment de-escalation for HPV-positive oropharyngeal cancer? A review of ongoing clinical trials

**DOI:** 10.3389/fonc.2022.1067321

**Published:** 2022-12-23

**Authors:** Emma A. Mensour, Shintha Alam, Seliya Mawani, Houda Bahig, Pencilla Lang, Anthony Nichols, David A. Palma, Katie Jasper

**Affiliations:** ^1^ Schulich School of Medicine & Dentistry, University of Western Ontario, London, ON, Canada; ^2^ Department of Radiology, Radiation Oncology and Nuclear Medicine, Université de Montréal, Montréal, QC, Canada; ^3^ Department of Radiation Oncology, London Health Sciences Centre, University of Western Ontario, London, ON, Canada; ^4^ Department of Otolaryngology-Head and Neck Surgery, University of Western Ontario, London, ON, Canada

**Keywords:** radiotherapy, chemoradiotherapy, human papillomavirus, oropharyngeal cancer, treatment de-escalation

## Abstract

**Background:**

Human papillomavirus (HPV)-associated oropharyngeal squamous cell carcinoma (OPSCC) has increased in incidence in recent decades. With higher cure rates in younger populations, long-term survivors may live with acute- and long-term toxicity, leading to increased interest in de-escalation treatment strategies for HPV-related OPSCC. Herein, we have examined the current landscape of clinical trials in this context.

**Methods:**

A review of active clinical trials related to de-escalation of HPV-associated OPSCC treatment was performed using the clinicaltrials.gov database from inception to January 2022. A search using the key words “oropharyngeal cancer” and “HPV” was completed. Three investigators independently reviewed each trial, with any discrepancies settled by a fourth. Data collected from each study included study phase, study design, primary, and secondary endpoints, and de-escalation treatment strategies. A final 24 articles were selected for full text review.

**Results:**

Many trials (n=19, 79%) were non-randomized, and most studies employed a phase II design (n=14, 58%). Only 13% (n=3) were randomized trials, and 8% (n=2) included a phase III component. The most frequent primary endpoint was progression-free survival (PFS) (n=9, 37.5%). With regards to the identified de-escalation strategies, all the studies (n=24) had at least one component assessing changes in RT dose/fractionation and/or a reduction in RT volumes. A smaller percentage of trials assessed surgical interventions (n=9, 37.5%) and/or changes in systemic therapy (n=8, 33.3%).

**Conclusion:**

A small number of randomized trials are underway, and a transition to more randomized phase III trials in the future will be important to change clinical practice.

## 1 Introduction

Between 2000 to 2012, the incidence of human papillomavirus (HPV)-associated oropharyngeal squamous cell carcinoma (OPSCC) increased worldwide, with reports of rising incidence in North America, United States, Europe, Japan, and Australia among others (1–3). Tobacco use and alcohol consumption have been historically considered the primary risk factors for head and neck cancers (2). Although the use of tobacco has decreased in recent years, the incidence of OPSCC continues to rise (2, 4) as a result of HPV-related cases. The subset of OPSCC groups with HPV is defined by the presence of high-risk HPV in tumor cells, primarily HPV type 16 (HPV 16) (5).

The treatment of HPV-associated OPSCC is evolving and depends on the stage and patient and physician preferences (6). The standard of care consists of a multidisciplinary approach that can include a combination of surgery, radiotherapy (RT), and systemic therapies. However, these strategies can result in substantial acute and long-term toxicities (7). These may include side effects such as dysphagia, lymphedema, mucositis, neurotoxicity, xerostomia, and rarely even death (8, 9). As a result, the potential to reduce toxicity and improve patient quality of life (QOL) using less-intensive treatment and other de-escalation strategies is garnering more attention (8).

Patients with HPV-related OPSCC tend to be diagnosed at a younger age and have significantly better prognosis and survival outcomes compared to HPV-negative OPSCC patients (5). Higher cure rates in a younger population can lead to a large group of longer-term survivors who must live with treatment-related toxicities for decades, underscoring the importance of treatment de-escalation. Common de-escalation strategies broadly include replacing, reducing, and omitting chemotherapy or RT, de-intensification of surgical resection through transoral surgery (TOS), with reduced-dose adjuvant RT, and/or induction chemotherapy followed by risk-adapted definitive therapy (8). Recent reviews have summarized completed de-escalation trials for patients with HPV-related OPSCC (10–12). In this article, we examine the current landscape of clinical trials in the context of treatment de-escalation for HPV-associated OPSCC patients, to increase clinician awareness of possible de-escalation strategies, and to help inform the design of additional de-escalation trials.

## 2 Methods

A review of active clinical trials related to de-escalation of HPV-associated OPSCC treatment for HPV-associated patients was performed using the *clinicaltrials.gov* database, searching from inception to January 2022. A search of actively recruiting trials using the key words “oropharyngeal cancer” and “HPV” was completed.

Studies were selected based on the following inclusion criteria:

- studies related to only HPV/p16-associated oropharyngeal cancer;
- studies testing de-escalation strategies;
- studies that are actively recruiting or not yet recruiting.

Exclusion criteria included:

- studies that were completed, withdrawn, terminated, or not recruiting;
- studies measuring efficacy of diagnostic procedures;
- studies evaluating novel vaccines as treatment for HPV;
- studies investigating biomarkers underlying HPV-associated OPSCC.

Data abstracted included study phase, study design, endpoints (e.g. progression-free survival [PFS], overall survival [OS], toxicity, QOL, disease-free survival [DFS], local control/locoregional control local recurrence, metastasis-free survival), RT dose/fractionation/volume, surgical interventions, and systemic therapy interventions. Three investigators independently reviewed each trial, with any discrepancies settled by a fourth.

## 3 Results

**Figure 1:**
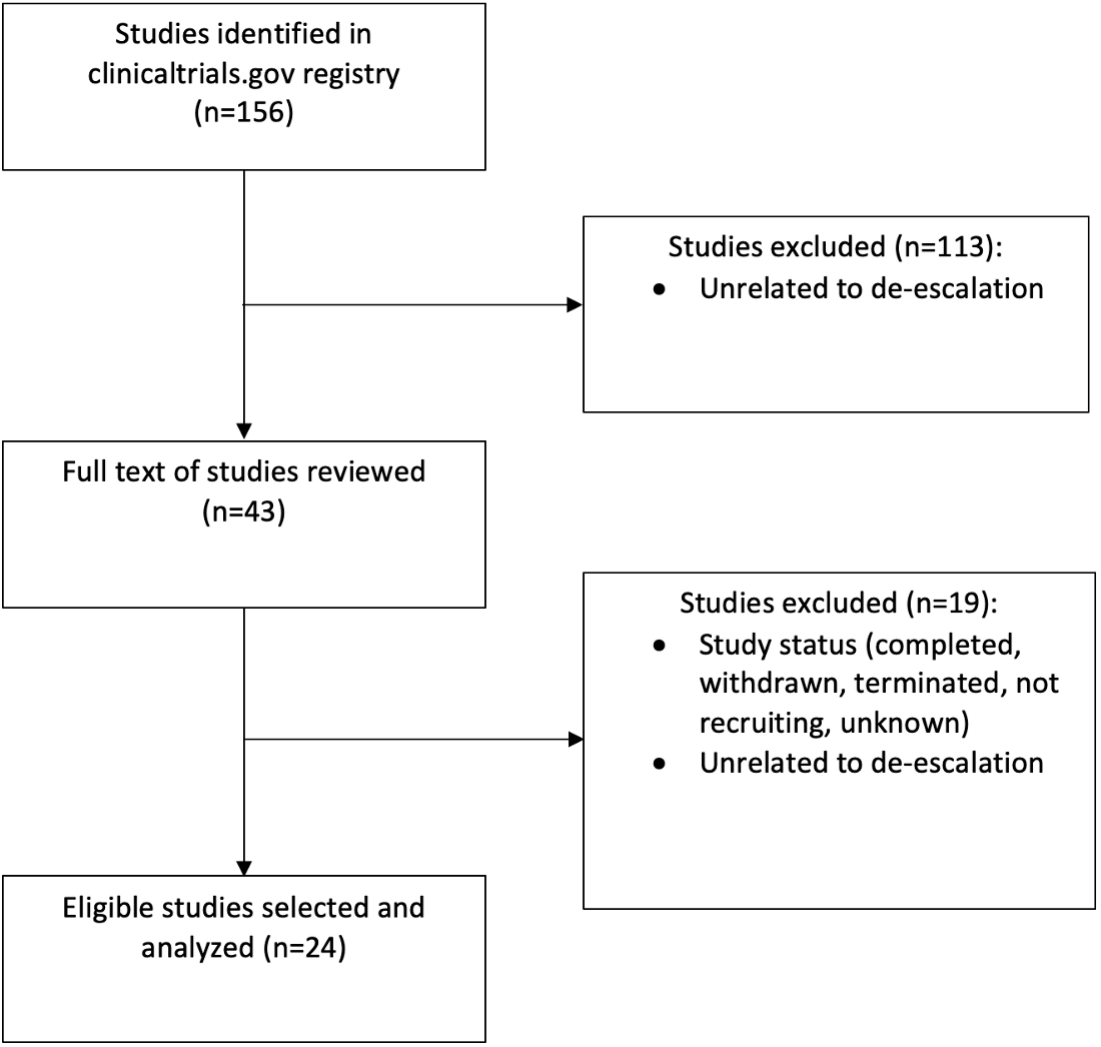
Search methodology utilized to identify eligible clinical trials investigating treatment de-escalation for patients with HPV- associated oropharyngeal cancer.

**Figure 2:**
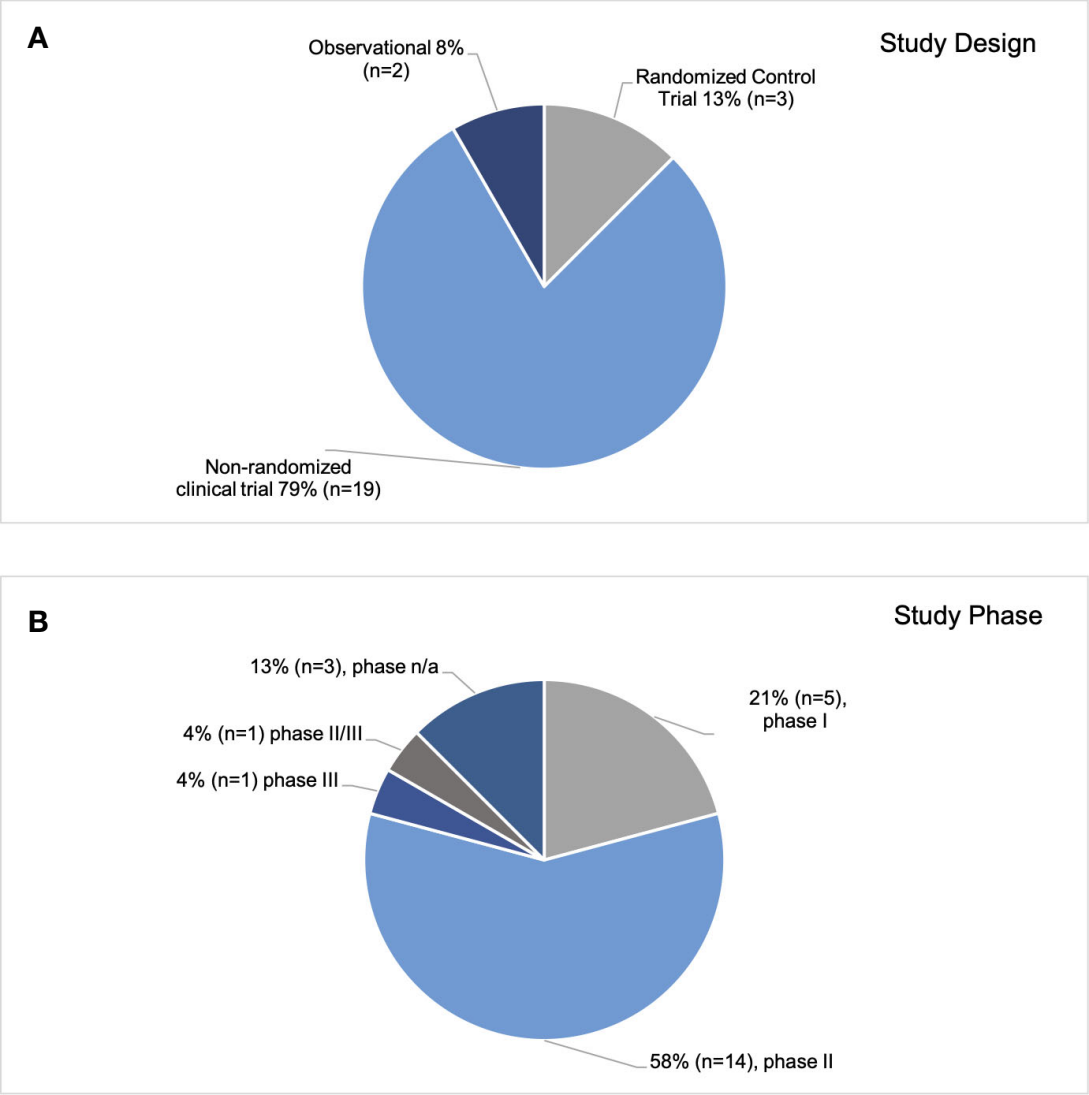
Study Design and Phase. Breakdown of analyzed trials by study design **(A)** and phase **(B)**.

Of the initial 156 clinical trials reviewed, a final 24 were selected based on a full text review (**Figure 1**) (13–36). Characteristics of the trials are shown in **Table 1**. The median sample size was 206 (range 24-1100). The most common study design was a non-randomized trial (n=19; 79%), followed by randomized control trials, (n=3, 13%), and observational studies (n=2, 8%) (**Figure 2**). Most studies (n=14, 58%) were phase II trials, while 21% (n=5) were phase I. Only two trials had a phase III component: one was a stand-alone phase III trial (PATHOS) and the other a phase II/III trial (NRG HN-005) design (**Figure 2**) (32). The most frequent primary endpoint (n=9, 37.5%) was PFS. Twenty-five percent (n=6) of the trials examined local control, locoregional control, or locoregional recurrence as a primary endpoint. The other primary endpoints, from most frequently measured to least, were toxicity (n=5), DFS (n=4), quality of life (n=3), OS (n=1) and other (complete response rate at six months) (n=1). OS was the most common secondary endpoint, measured in 83% (n=20) of studies. Seventy-nine percent (n=19) and 42% (n=10) of the trials examined toxicity and PFS as secondary endpoints, respectively. Thirty-eight percent (n=9) of trials measured local control and quality of life. The least frequent secondary endpoints studied were disease-free survival (25%, n=6), metastasis-free survival, and other (circulating tumour DNA levels, and incidence of acute grade ≥3 or higher functional mucosal adverse events) (both 8%, n=2).

**Table 1:** Summary of analyzed study characteristics.

	Number of Studies (n)	% Of Total Studies (n = 24)
Study Phase		
I	5	21%
II	14	58%
III	1	4%
II/III	1	4%
N/A	3	13%
Study Design		
Randomized Control Trial	3	13%
Non-randomized Control Trial	19	79%
Observational	2	8%
Primary Endpoints*		
Progression-free Survival	9	38%
Overall Survival	1	4%
Toxicity	5	21%
Quality of Life	3	13%
Disease-free Survival	4	17%
Local Control/Locoregional Control/Local Recurrence	6	25%
Other	1	4%
Secondary Endpoints*		
Progression-free Survival	10	42%
Overall Survival	20	83%
Toxicity	19	79%
Quality of Life	9	38%
Disease-free Survival	6	25%
Metastasis-free Survival	2	8%
Local Control/Locoregional Control/Local Recurrence	9	38%
Other	2	8%
De-escalation Strategies*		
Radiotherapy Dose/Fractionation/Volume	24	100%
Surgical Intervention	9	37.5%
Systemic Therapy	8	33.3%

*Several studies examined more than one primary endpoint, secondary endpoint, or de-escalation strategy.

With regards to the identified de-escalation strategies, all the studies (n=24) assessed changes in RT dose/fractionation and/or a reduction in RT volumes. A smaller percentage of trials assessed surgical interventions (n=9, 37.5%) and systemic therapy (n=8, 33.3%). Several individual trials examined more than one of these three strategies, with three trials examining all three.

A detailed summary of each of the reviewed trials is provided in **Tables 2**–**5**, and the following sections discuss some of the de-escalation approaches.

**Table 2:** Current trials examining radiotherapy de-escalation for treatment of HPV-associated OPSCC.

Trial, Accrual Dates	Trial Design (Sample Size)	Overall Stage	De-escalation Strategy			Primary Endpoint
			Radiotherapy	Surgery	Systemic Therapy	
De-escalation Strategy: Radiotherapy Alone						
NCT03777384 (13), 2018-2023	Prospective, Observational, Phase I (n=60)	Stage I-II (AJCC 8th ed.)	De-escalated RT (not stated)	N/A	N/A	Local control, DFS
ReACT (NCT04900623) (14), 2021-2030	Phase II (n=145)	Stage I-III (AJCC 8th ed.)	RT 70 Gy/35 vs de-escalated dose (not stated) +/- chemotherapy	N/A	N/A	2-year PFS
NCT03215719 (15), 2017-2021	Phase II (n=54)	Stage III-IVa (AJCC 7th ed.)	CRT 70 Gy/35 vs de-escalated dose (not stated)	N/A	N/A	2-year PFS
NCT04667585 (16), 2020-2027	Interventional (n=120)	Stage I-III (AJCC 8th ed.)	CRT 70 Gy/35 vs de-escalated dose based on mid treatment response in lymph node volume.	N/A	N/A	2-year PFS
HYHOPE (NCT04580446) (17), 2020-2024	Phase I (n=24)	Stage I-II (AJCC 8th ed.)	*CRT 46.6 Gy/15 vs CRT 52 Gy/20, reduction in elective nodal volume size	N/A	N/A	Toxicity
ENID (NCT04444869) (19), 2020-2024	Phase II (n=28)	Stage I-II (AJCC 8th ed.)	*Reduced RT dose to elective nodal volume	N/A	N/A	Rate of PEG tube placement.
NCT03323463 (20), 2017-2022	Phase II (n=300)	Stage III-IVa (AJCC 7th ed.)	CRT 70 Gy/35 vs CRT 30 Gy/15	N/A	N/A	N/A
NCT04178174 (21), 2019-2025	Randomized Phase II (n=106)	Stage I-II (AJCC 8th ed.)	*CRT 70 Gy/35 vs CRT 40 Gy/20 + SBRT 14 Gy/2 boost	N/A	N/A	2-year LRC
NCT03799445 (22), 2019-2022	Phase II (n=180)	Stage I-II (AJCC 8th ed.)	Ipilimumab + nivolumab + RT 60 Gy/30	N/A	N/A	2-year PFS, toxcitiy, 6-month complete response rate
NCT03396718 (23), 2018-2026	Phase I (n=384)	Stage II-III (AJCC 8th ed.)	PO(C)RT 60-66 Gy vs PO(C)RT 48.8-55 Gy	N/A	N/A	24-month LRC

PORT, post-operative radiotherapy; TOS, trans-oral surgery (with neck dissection); POCRT, post-operative chemoradiotherapy; OS, overall survival; MDADI, MD Anderson dysphasia inventory; PFS, progression free survival; RT, radiotherapy; DFS, disease free survival; Fx, fraction; TORS, trans-oral robotic surgery (with neck dissection); SBRT, stereotactic body radiotherapy. *Trial with RT volume reduction.

N/A, Not applicable.

**Table 3:** Current trials examining radiotherapy de-escalation and surgery for treatment of HPV-associated OPSCC.

Trial, Accrual Dates	Trial Design (Sample Size)	Overall Stage	De-escalation Strategy			Primary Endpoint
			Radiotherapy	Surgery	Systemic Therapy	
De-escalation Strategy: Radiotherapy + Surgery						
NCT04638465 (25), 2020-2025	Prospective, Observational (n=1000)	Stage I-III (AJCC 8th ed.)	CRT 70 Gy/35 vs CRT 60 Gy/30	TORS alone vs TORS + chemo	N/A	10-year DFS, Toxicity
NCT04920344 (26), 2021-2022	Phase II (n=40)	Stage I-III (AJCC 8th ed.)	PORT 50 Gy/25 vs POCRT 60 Gy/30	TOS alone vs TOS + PO(C)RT	N/A	MDADI at 24 weeks and 1-year, QoL
NCT05119036 (27), 2021-2024	Phase II (n=75)	Stage I-II (AJCC 8th ed.)	PORT 54 Gy/27 vs PORT 44 Gy/22	TORS for all	N/A	2-year DFS
NCT04277858 (28), 2020-2024	Phase II (n=60)	Stage III-IVa (AJCC 7th ed.)	PORT for salvage only	Induction chemo; TORS +/- PORT	N/A	2-year PFS
NCT03729518 (29), 2018-2024	Phase II (n=150)	Stage III-IVa (AJCC 7th ed.)	*PORT 50 Gy/25, contralateral dose reduced to 45 Gy	TORS alone vs TORS + PORT	N/A	2-year LRC
NCT04489212 (30), 2020-2025	Phase I (n=45)	Stage III-IVb (AJCC 8th ed.)	*PORT vs mucosal sparing PORT	TORS for all	N/A	2-year LC

PORT, post-operative radiotherapy; TOS, trans-oral surgery (with neck dissection); POCRT, post-operative chemoradiotherapy; OS, overall survival; MDADI, MD Anderson dysphasia inventory; PFS, progression free survival; RT, radiotherapy; DFS, disease free survival; Fx, fraction; TORS, trans-oral robotic surgery (with neck dissection); SBRT, stereotactic body radiotherapy. *Trial with RT volume reduction.

N/A, Not applicable.

**Table 4:** Current trials examining radiotherapy and chemotherapy de-escalation for treatment of HPV-associated OPSCC.

Trial, Accrual Dates	Trial Design (Sample Size)	Overall Stage	De-escalation Strategy			Primary Endpoint
			Radiotherapy	Surgery	Systemic Therapy	
De-escalation strategy: Radiotherapy + Chemotherapy						
Quarterback 2B (NCT02945631) (33), 2016-2027	Phase II (n=50)	Stage III or IV (AJCC 8th ed.)	Induction chemo; CRT 56 Gy/28 vs CRT 50.4 Gy/28	N/A	Induction chemo; low dose CRT (not stated dose/agent)	3 & 5-year PFS
NCT03952585 (34), 2019- 2025	Randomized Phase II/III (n=711)	Stage I-II (AJCC 8th ed.)	CRT 35 fx (dose not stated) vs CRT 30 fx (dose not stated) vs nivolumab + RT 30 fx (dose not stated)	N/A	Concurrent cisplatin vs nivolumab	6-year PFS, QoL
NCT04867330 (35), (2021-2026)	Interventional (n=46)	Stage I-III (AJCC 8th ed.)	Induction chemo; CRT 70 Gy/35 vs RT 60 Gy/30	N/A	Induction chemo (toripalimab + docetaxel + cisplatin); CRT 70 Gy/35 with cisplatin vs RT 60 Gy/30	2-year PFS
NCT03875716 (36), 2019-2027	Phase II (n=111)	Stage I (AJCC 8th ed.)	PORT 60 Gy vs PORT 46 Gy vs observation	N/A	PORT 60 Gy without chemotherapy	2-year DFS
NCT03416153 (18), 2018-2022	Phase II (n=75)	Stage I-II (AJCC 8th ed.)	CRT 70 Gy/35 vs CRT 54 Gy (not stated fractions) based on mid treatment PET response.	N/A	Concurrent carboplatin and paclitaxel	1-year LRR

PORT, post-operative radiotherapy; TOS, trans-oral surgery (with neck dissection); POCRT, post-operative chemoradiotherapy; OS, overall survival; MDADI, MD Anderson dysphasia inventory; PFS, progression free survival; RT, radiotherapy; DFS, disease free survival; Fx, fraction; TORS, trans-oral robotic surgery (with neck dissection); SBRT, stereotactic body radiotherapy. *Trial with RT volume reduction.

N/A, Not applicable.

**Table 5:** Current trials examining radiotherapy and chemotherapy de-escalation and surgery for treatment of HPV-associated OPSCC.

Trial, Accrual Dates	Trial Design (Sample Size)	Overall Stage	De-escalation Strategy			Primary Endpoint
			Radiotherapy	Surgery	Systemic Therapy	
De-escalation Strategy: Radiotherapy + Surgery+ Chemotherapy						
NCT04502407 (31), 2020-2025	Phase II (n=36)	Stage I-II (AJCC 8th ed.)	POCRT 50 Gy/25 vs POCRT 30 Gy/15	TORS for all	POCRT 50 Gy/25 with 5 cycles cisplatin 40 mg/m^2^) vs 3 cycles	2-year PFS
NCT04572100 (24), 2020-2023	Phase I (n=36)	Stage II-III (AJCC 8th ed.)	Induction chemo; CRT 70 Gy/35 vs CRT 25 fx (dose not stated) vs RT 25 fx (dose not stated)	Induction chemo; TORS alone vs TORS + PO(C)RT	Induction Chemo (carboplatin and Paclitaxel) for all	N/A
PATHOS (NCT02215265) (32), 2014-2027	Randomized Phase III (n=1100)	Stage I-II (AJCC 8th ed.)	Low risk: No PORT Int Risk PORT 60 Gy/30 vs PORT 50Gy/25 High Risk: PORT 60Gy/30 vs POCRT 60Gy/30	TORS for all and risk stratification based on pathological features	High Risk only: PORT 60 Gy/30 vs POCRT 60 Gy/30	OS, MDADI at 12-months, and QoL

PORT, post-operative radiotherapy; TOS, trans-oral surgery (with neck dissection); POCRT, post-operative chemoradiotherapy; OS, overall survival; MDADI, MD Anderson dysphasia inventory; PFS, progression free survival; RT, radiotherapy; DFS, disease free survival; Fx, fraction; TORS, trans-oral robotic surgery (with neck dissection); SBRT, stereotactic body radiotherapy. *Trial with RT volume reduction.

N/A, Not applicable.

All the eligible clinical trials assessed changes in RT dose/fractionation and/or a reduction in RT volumes (n=24). De-escalation may involve reduction in RT biologically effective dose (BED) or volume as well as the reduction or omission of chemotherapy. For example, the approach of the Quarterback 2b phase II clinical trial (NCT02945631) is to reduce the RT dose after induction chemotherapy (33). In this study, RT is reduced to 56 Gy in 28 fractions (arm I) and 50.4 Gy in 28 fractions (arm II) from the standard 70 Gy in 35 fractions (33). The phase II study is expected to recruit 50 participants and aims to determine the safety and efficacy of reduced dose RT in patients with advanced stage disease (stages III or IV, AJCC 8^th^ edition). The trial is still ongoing and is evaluating the progression free survival (PFS) and quality of life of patients with early stages of HPV-associated OPSCC (stage I and II, AJCC 8^th^ edition).

Furthermore, a phase II randomized control trial conducted by the Centre Hospitalier de l’Université de Montréal (NCT04178174) is utilizing a stereotactic body radiotherapy (SBRT) boost alongside a shortened course of RT (21). SBRT is followed by de-escalated chemoradiotherapy (CRT) (40 Gy in 20 fractions with concurrent cisplatin) compared to the standard CRT (70Gy in 33 fractions with concurrent cisplatin). Additionally, a single arm Phase I study (NCT04580446) by the University of Texas Southwestern Medical Center is evaluating the efficacy and tolerability of de-intensified hypo-fractionated RT for HPV-associated OPSCC and reduction in effective nodal dose (17). Hypofractionated RT will be delivered to a BED of 60 Gy (compared to the current standard of 70 Gy). This will involve 46.5 Gy in 15 fractions with weekly cisplatin (40 mg/m^2^)). The elective nodal volume irradiated will be restricted to involve nodal levels and one immediately adjacent level. However, if tolerability of the 3-week regimen is poor, 52 Gy in 20 fractions will be utilized.

Nine ongoing clinical trials are currently investigating the use of TOS/TORS with or without concurrent or adjuvant RT/CRT in the treatment de-intensification of HPV-associated OPSCC (n=9, 37.5%).

As an example, the largest such study is the PATHOS trial (NCT02215265) (32). This trial is a large, randomized phase III trial with a target accrual of 1100 patients from 41 different institutions. The trial aims to optimize patient outcomes by reducing the intensity of adjuvant treatment protocols for HPV-associated OPSCC after TORS. Patients with low risk pathological features (T1-2, 0-1 node <3cm, negative margins, no PNI or LVI, and no extracapsular spread (ECS)) will be observed. Patients with intermediate risk disease (T3, close margins, multiple nodes, PNI, LVI, but no ECS) will be randomized to one of two PORT regiments (60 Gy in 30 fractions and 50 Gy in 25 fractions). Patients with high risk pathology (positive margins or ECS) will be randomized to PORT 60 Gy in 30 fractions with and without concurrent cisplatin. The study also aims to look at secondary outcomes, such as whether swallowing function can be improved, and acute and late toxicities.

## 4 Discussion

Our review of active de-escalation trials has yielded several important insights. The studies evaluating the use of altered radiotherapy regimens, chemotherapy regimens, and primary surgery followed by reduced adjuvant therapy as de-escalation strategies for HPV-associated OPSCC demonstrate important prospective efforts that are underway to help guide the shared decision-making process for treating this patient population. These ongoing clinical trials have the commonality of assessing changes in RT dose/fractionation and/or a reduction in RT volumes with a smaller percentage examining primary surgery and systemic therapy as de-escalation strategies as well. If these de-escalation approaches are successful, acute, and long-term side effects could potentially be reduced, resulting in improved quality of life for patient with HPV- associated OPSCC.

Although several studies and clinical trials identify potential novel de-escalation strategies for HPV- associated OPSCC, there are important study limitations to consider. Firstly, there are few studies that are randomized, which may lead to bias when comparing the results against other modalities. However, randomized control trials are not always feasible due to logistical difficulties, cost, and unwillingness for patients or physicians to enroll which results in difficulty in accrual and small sample sizes. For example, one of the most contentious issues in the field is whether to manage these patients with primary surgery or primary radiation. The randomized studies on this topic have either failed to accrue (NCT03691441, PMID 32727416) or have been modest in sample size (PMID 35482348, 31416685) (11, 37).

While there are several ongoing phase I and II trials, phase III data is needed to adopt these de-escalation strategies as standard of care. Among the studies included, there was only one phase III trial and one phase II/III ongoing trial. As a result, there may be very limited practice-changing data available in the next five years.

In recent years, there has been ongoing research appraising the use of circulating HPV DNA (HPV ctDNA) to serve as a residual tumor marker at the end of chemoradiation or to predict relapse during follow up period (38). The use of de-escalation strategies and blood-based biomarkers for HPV-associated OPSCC warrant further investigation for informing future management.

In light of the rapidly-changing portfolio of trials examining this issue, a ‘living systematic review’ that is continually updated may be useful in the future.

### 4.1 Limitations of this Study

The studies included in this paper were accessed only through clinicaltrials.gov. No other platforms or databases were accessed, and therefore it is possible that studies listed on other registries were missed. In addition, studies were retrieved from the clinicaltrials.gov registry in January 2022 and we only reviewed trials that were active and recruiting, or pending recruiting, during the study period. There is a possibility that studies relevant to the search criteria were added after January 2022 and were therefore unable to be included in the paper. However, there are other studies that did not meet our inclusion criteria (e.g. completed accrual before our inclusion period, or added to clinicaltrials.gov after the search was completed) including the DART-HPV study (NCT02908477) (39) and the EVADER study (NCT03822897) (40). The DART-HPV study is a phase III clinical trial evaluating de-escalated CRT (30 Gy in 20 fractions with concurrent docetaxel) compared to the standard CRT (60Gy in 30 fractions with concurrent cisplatin). Similarly, the EVADER study is a phase II single arm trial evaluating de-escalated CRT. Furthermore, the data fields available on clinicaltrials.gov are limited, and may not reflect all the nuances of treatment that are more fully described in the full protocols. For example, we may have not captured adaptive radiotherapy trials where radiation volumes are adapted based on response, without dose reduction. Finally, there is a possibility that studies relevant to the search criteria had completed participant accrual and therefore were unable to be included in the paper.

## 5 Conclusion

Based on the characteristics of the studies analyzed, it is possible that several therapeutic de-escalation strategies could be as effective standards of care in the future, most of which are based on changes in RT approaches. Following the completion of these phase II trials, phase III trials may be needed to generate sufficient evidence to change practice.

## Data availability statement

Publicly available datasets were analyzed in this study. This data can be found here: www.clinicaltrials.gov.

## Author contributions

These authors EM, SA, and SM contributed equally to this work and share first authorship. All authors contributed to the article and approved the submitted version.
